# The Yin and Yang of vitamin D receptor (VDR) signaling in neoplastic progression: Operational networks and tissue-specific growth control

**DOI:** 10.1016/j.bcp.2009.09.005

**Published:** 2010-01-01

**Authors:** F.C. Campbell, Haibo Xu, M. El-Tanani, P. Crowe, V. Bingham

**Affiliations:** Centre for Cancer Research and Cell Biology, Queen's University of Belfast, Lisburn Rd, Belfast BT9 7BL, Northern Ireland, UK

**Keywords:** 1α,25-(OH)_2_ D_3_, one alpha, 25 dihydroxyvitamin D3, APC, adenomatous polyposis coli, CRC, colorectal cancer, DMBA, dimethylbenzanthracene, DR3-type, directly repeated arrangement of the hexameric binding sites with three spacing nucleotides, DRIP, Vitamin D receptor-interacting protein, ERK, extracellular signal-regulated kinase, GSK3β, glycogen synthase kinase beta, HDAC, histone deacetylator co-repressor complex, MAPK, mitogen-activated protein kinase, NCoR, nuclear receptor co-repressor, NHL, non-Hodgkins lymphoma, OPN, osteopontin, RAC3, receptor activated coactivators 3, ROCK, Rho-associated coiled kinase, RXR, retinoid X receptor, SRC-1, steroid receptor coactivators-1, Tcf, T cell factor, TIF2, transcriptional intermediary factor 2, TPA, 12-O-tetradecanoylphorbol-13-acetate, VDRE, vitamin D response element, VDR, vitamin D receptor, WINAC, Williams syndrome transcription factor (WSTF) including nucleosome assembly complex, 1α,25-(OH)_2_ D_3_, Vitamin D receptor, Signaling, Cancer

## Abstract

Substantive evidence implicates vitamin D receptor (VDR) or its natural ligand 1α,25-(OH)_2_ D_3_ in modulation of tumor growth. However, both human and animal studies indicate tissue-specificity of effect. Epidemiological studies show both inverse and direct relationships between serum 25(OH)D levels and common solid cancers. VDR ablation affects carcinogen-induced tumorigenesis in a tissue-specific manner in model systems. Better understanding of the tissue-specificity of vitamin D-dependent molecular networks may provide insight into selective growth control by the seco-steroid, 1α,25-(OH)_2_ D_3_. This commentary considers complex factors that may influence the cell- or tissue-specificity of 1α,25-(OH)_2_ D_3_/VDR growth effects, including local synthesis, metabolism and transport of vitamin D and its metabolites, vitamin D receptor (VDR) expression and ligand-interactions, 1α,25-(OH)_2_ D_3_ genomic and non-genomic actions, Ca^2+^ flux, kinase activation, VDR interactions with activating and inhibitory vitamin D responsive elements (VDREs) within target gene promoters, VDR coregulator recruitment and differential effects on key downstream growth regulatory genes. We highlight some differences of VDR growth control relevant to colonic, esophageal, prostate, pancreatic and other cancers and assess the potential for development of selective prevention or treatment strategies.

## Graphical abstract

**Figure:**
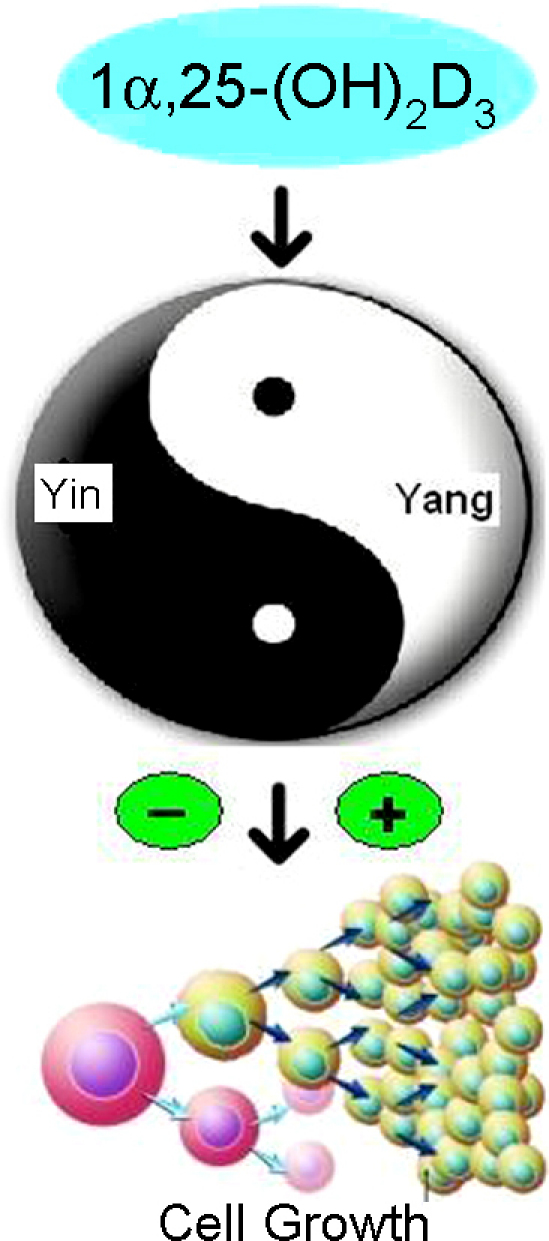

## Keypoints

• Complex factors influence the cell- or tissue-specificity of vitamin D biological and growth effects, including local synthesis, metabolism and transport of vitamin D and its metabolites, vitamin D receptor (VDR) expression and ligand-interactions, 1α,25-(OH)_2_ D_3_ genomic and non-genomic actions, 1α,25-(OH)_2_ D_3_-mediated Ca^2+^ flux, kinase activation, VDR interactions with specific vitamin D responsive elements within target gene promoters, VDR coregulator recruitment and differential effects on key downstream target genes.
• Animal and in vitro studies show cell- or tissue-restricted vitamin D growth control.
• Epidemiological studies indicate vitamin D tissue-specific effects on neoplastic progression.
• E-cadherin and osteopontin (OPN) are functionally antagonistic VDR target genes that orchestrate the growth response to 1α,25-(OH)_2_ D_3_ in diverse tumor types.
• Consideration of 1α,25-(OH)_2_ D_3_-dependent signaling networks in a cell-lineage or tissue-specific context may shed light on its disparate growth regulatory effects and help exploit the promising therapeutic potential of VDR ligands, for selected cancers.

## 1 Introduction

### 1.1 The vitamin D endocrine system

Groundbreaking discoveries of the early 20th century elucidated vitamin D's essential role in calcium and phosphate homeostasis, bone mineralization and enabled major public health advances. The capacity of a novel fat-soluble vitamin that was distinct from vitamins A, B or C, for prevention of experimental Rickets was shown in a seminal study by Mellanby [1]. Subsequently, Chick et al. demonstrated that clinical Rickets could be cured by dietary cod liver oil supplementation or sunlight exposure [2]. The Nobel prize in Chemistry was awarded to Dr Adolf Windaus in 1928, in recognition of his achievement in isolation of vitamin D and demonstration of its steroid structure [3]. In the 1930s, fortification of milk with vitamin D virtually eradicated Rickets from the United States, although it had previously been a highly prevalent crippling disease of childhood [4].

The mammalian form of vitamin D is a fat-soluble prohormone cholecalciferol (vitamin D_3_) that may be generated endogenously by ultraviolet light-mediated metabolism of the precursor sterol 7-dehydrocholesterol, in the skin. Alternatively, vitamin D_3_ may be obtained from dietary sources [5]. This prohormone (cholecalciferol) is hydroxylated to 25-hydroxycholecalciferol (25(OH)D_3_) by hepatocyte 25-hydroxylase. Further hydroxylation by 1α-hydroxylase (CYP27B1), into the main biologically active hormone, 1α 25-dihydroxycholecalciferol (1α,25-(OH)_2_ D_3_ or calcitriol) occurs in the proximal renal tubule in a tightly regulated fashion [6]. 1α,25-(OH)_2_ D_3_ then acts as a steroid chemical messenger in a diverse target tissues, in what is known as the vitamin D endocrine system [7]. To meet needs of bone mineralization, 1α,25-(OH)_2_ D_3_ stimulates intestinal calcium and phosphate absorption, bone calcium and phosphate metabolism as well as renal calcium and phosphate reabsorption, by differential effects on osteoblasts, chondrocytes, renal and intestinal epithelia [8]. Furthermore, discovery of VDR expression in diverse normal human tissues including B and T lymphocytes, the hair follicle, muscle, adipose tissue, bone marrow and in cancer cells has widened the perceived scope of the vitamin D endocrine system, beyond bone homeostasis [7].

### 1.2 1α,25-(OH)_2_ D_3_ chemical structure and conformational relationships

**Fig. 1:**
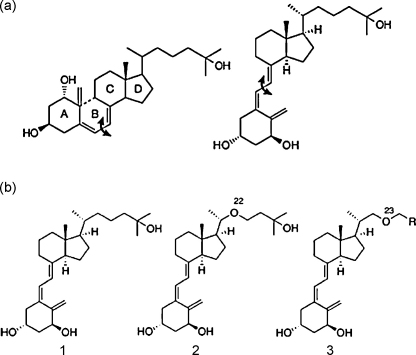
Chemistry of 1α,25-(OH)_2_ D_3_. 1α,25-(OH)_2_ D_3_ is derived from the 4 cyclopentanoperhydro-phenanthrene ring structure (A, B, C, and D rings) for steroids. In 1α,25-(OH)_2_ D_3_, the 9,10 carbon–carbon bond of ring B is broken between ring A and rings C and D (arrow, a) and the molecule is technically classified as a seco-steroid. The molecule may then rotate along the bond between ring A and rings C and D (arrow), to provide the structure of 1α,25-(OH)_2_ D_3_ (a). Stepwise modification of the molecule, involving location of a oxygen atom at position 23 on the C and D ring side chain or removal of the terminal –OH group can have important biological effects (b).

1α,25-(OH)_2_ D_3_, the active form of vitamin D, is a highly flexible molecule with a steroid carbon skeleton, involving 4 fused cyclopentanoperhydro-phenanthrene rings, A–D. Unlike other steroids, the 9–10 carbon bond is broken, thus creating a conformationally flexible molecule in which the “A” ring may rotate (Fig. 1). The molecule is technically classified as a seco-steroid. The spatial arrangements of principal functional components of the 1α,25-(OH)_2_ D_3_ molecule comprise a hugely important determinant of its biological activities. *Cis*–*trans* isomerism influences stability and reactivity. The unusual degree of flexibility within 1α,25-(OH)_2_ D_3_ enables synthesis of structural analogs (Fig. 1b) that elicit well-defined subsets of the vitamin D response (see below) [9,10].

### 1.3 Vitamin D transport

Normally, only 0.04% of 25(OH)D and 0.4% of 1α,25-(OH)_2_ D_3_ are free in plasma, the remainder being tightly bound to either a vitamin D transporter protein (DBP) (85–88%; high affinity; dissociation constant [*K*_d_] ∼ 1 nM) [11] or albumin (12–15%; low affinity) [12]. Only free unbound vitamin D sterols are considered to be biologically active, since only the free form and not DBP-bound 1α,25-(OH)_2_ D_3_ induces metabolic responses in target cells [13]. In addition to transport, DBP functions to maintain stable serum stores of vitamin D metabolites, modulate bioavailability and influence responsiveness of some end-organs [14]. 1α,25-(OH)_2_ D_3_ binds to its “nuclear” receptor (VDR) with high affinity (dissociation constant value of [*K*_d_] ∼ 1 nM or lower) [15].

### 1.4 The vitamin D receptor (VDR)

**Fig. 2:**
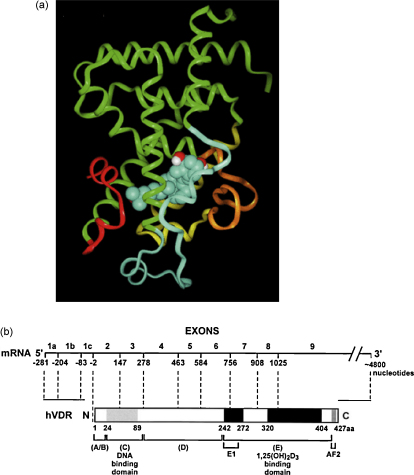
Schematic representation of the vitamin D receptor (VDR) domain structure. (a) VDR protein backbone and 1α,25-(OH)_2_ D_3_ ligand-binding pocket. The VDR protein backbone is represented by a ribbon. A space-filling representation of 1α,25-(OH)_2_ D_3_ is shown within the VDR ligand-binding pocket by an atom-based structure. Conformational modification of the vitamin D side chain may influence ligand binding and transcriptional activity [125]. (b) The human *VDR* gene domain structure. The human *VDR* gene is composed of 9 exons that encode domains (A–F). Upon 1α,25(OH)_2_ D_3_ binding to the hormone ligand-binding domain, VDR is stabilized by the phosphorylation of serine 51 in the DNA-binding domain and serine 208 in the hinge region. VDR associates with the retinoic acid receptor (RXR) through the dimerization domains in E/F. The 1α,25-(OH)_2_ D_3_–VDR–RXR complex binds to the vitamin D response elements (VDREs) through the DNA-binding domain in the promoters of target genes. Conformational change in VDR results in co-repressor dissociation and enables interaction of the AF2 transactivation domain with stimulatory coactivators, such as steroid receptor coactivators (SRCs), vitamin D receptor-interacting proteins complex and nuclear coactivators.

Free 1α,25-(OH)_2_ D_3_ enters the cell and binds the vitamin D receptor (VDR) (Fig. 2a), that may be present in the cytoplasm, nucleus or partitioned between the cytoplasm and nucleus [16]. VDR is an endocrine member of the nuclear receptor superfamily [8] with high structural and ligand-binding homology across various species [6]. Ligands for VDR include bile acid metabolites as well as 1α,25-(OH)_2_ D_3_ [17]. VDR has the same modular structure as other members of the nuclear receptor superfamily, including an N-terminal A/B region, a conserved DNA-binding domain, a flexible hinge region and a moderately conserved ligand-binding pocket that contains a dimerization interface and a ligand-dependent transcriptional activation domain, AF-2 [18] (Fig. 2a and b). Ligand binding induces a conformational change of the AF-2 region that allows dissociation of accessory proteins, exposure of the DNA-binding pocket and recruitment of coactivators [19]. Specific mutations that cause deletions, frameshift mutations, premature stop codons or splice site abnormalities that impede VDR expression or binding activity, effectively suppress key VDR actions [20].

### 1.5 1α,25-(OH)_2_ D_3_/VDR mediated genomic responses

The 1α,25-(OH)_2_ D_3_/VDR complex functions to regulate gene transcription through heterodimerization with any of three retinoid X receptor (RXR) isoforms and binds to cognate vitamin D responsive elements (VDREs) in the promoter region of target genes. VDRE structures within promoter regions of primary 1α,25-(OH)_2_ D_3_ regulated genes can vary [21]. However, the majority of known VDREs show a DR3-type structure comprising a directly repeated arrangement of hexameric binding sites with 3 spacing nucleotides [22]. This arrangement provides the most efficient interface for VDR/RXR heterodimer binding to core VDREs. Subclasses of DR3 VDREs show some sequence variation but their in vivo functionality is proportional to their in vitro binding affinity for VDR–RXR heterodimers [23]. Strongest affinity has been observed among class I DR3-type VDREs, including that present in the osteopontin (OPN) promoter [23]. 1α,25-(OH)_2_ D_3_ may regulate genes that do not contain VDREs within their promoter regions, through non-genomic VDR actions (see below).

### 1.6 VDR/VDRE mediated coactivation or corepression of gene transcription

Regulation of gene expression by 1α,25-(OH)_2_ D_3_ genomic signaling, is dependent upon the ability of VDR/RXR heterodimers to recruit coregulatory protein complexes [24] that may activate or repress target gene expression. Ligand triggered conformational change of VDR–RXR heterodimers results in dissociation of co-repressor proteins such as NCoR (nuclear receptor co-repressor) and facilitates the interaction with members of the CBP/p300 and p160 coactivator families including SRC-1 (steroid receptor coactivators-1), TIF2 (transcriptional intermediary factor 2), and RAC3 (receptor activated coactivators 3) [25]. DRIP (vitamin D receptor-interacting protein) cofactor complexes are also involved in parallel [19]. These coactivators bind ligand-activated VDR, induce a coactivator exchange in the transcriptional complex of VDR-responsive promoters [26] and enables opening of the chromatin structure. These effects create an environment suitable for gene transcription [27].

**Fig. 3:**
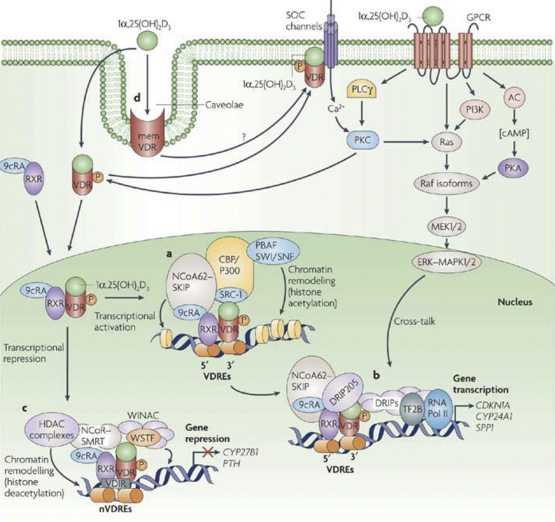
Transcriptional regulation by vitamin D. Classical action of 1α,25-(OH)_2_ D_3_ is mediated by binding of the VDR/RXR complex at VDREs. (a) Transcriptional activation involves the co-activator molecules SRCs, NCoA62, HATs, CBP p300 and other molecules to derepress chromatin. (b) Binding of DRIP to the AF2 domain attracts a complex containing transcription factor 2B (TF2B) and RNA polymerase II (RNA Pol II) for transcription initiation. The presence of the multiprotein complex facilitates increased gene transcription. (c) 1α,25(OH)_2_ D_3_-mediated transcriptional repression involves VDR–RXR heterodimer association with VDR-interacting repressor (VDIR) bound to E-box-type negative VDREs (nVDREs), dissociation of the HAT co-activator and recruitment of histone deacetylase (HDAC) co-repressor. Williams syndrome transcription factor (WSTF) potentiates transrepression by interacting with a multifunctional, ATP-dependent chromatin-remodelling complex (WINAC) and chromatin. This leads to the repression of genes, such as *CYP27B1* (which encodes 1α-OHase) and *PTH* (which encodes parathyroid hormone). (d) Non-genomic, rapid actions of 1α,25-(OH)_2_ D_3_ are though activation of mitogen-activated protein kinase (MAPK)–extracellular signal-regulated kinase (ERK) 1 and 2 cascade through the phosphorylation (P) and activation of Raf by protein kinase C (PKC), partly through induced changes of intra-cellular Ca^2+^ concentration (reproduced from Deeb et al. [126] with kind permission of Dr CS Johnston Roswell Park CI, New York and the publisher).

VDR may also repress gene transcription. CYP27B1 catalyzes the metabolic activation of 1α,25-(OH)_2_ D_3_ from its precursor [28] and is negatively regulated by 1α,25-(OH)_2_ D_3_, in a cell-lineage-specific and tissue-restricted manner [29]. CYP27B1 and other genes including PTH [30] are thought to be suppressed by 1α,25-(OH)_2_ D_3_ via negative vitamin D response elements (VDREs). Ligand-activated VDR binds to the 5’ half site of negative VDREs [30]. VDR–RXR heterodimers interact with a VDR-interacting repressor (VDIR) at the E-box type element of negative VDREs, comprising a CANNTG-like motif. Such interactions at the E-box induce coregulator switching, involving dissociation of p300 coactivators but association of the histone deacetylator (HDAC) co-repressor complex, resulting in ligand-induced transrepression. Cell or tissue-specific transrepression of CYP27B1 by 1α,25-(OH)_2_ D_3_ may involve multiple VDREs located in more distal promoter regions that enhance chromatin looping and interactions with protein super complexes of differing transcriptional abilities. Other mechanisms of transrepression involve the association between WINAC (Williams syndrome transcription factor (WSTF) including nucleosome assembly complex), a multifunctional, ATP-dependent chromatin-remodelling complex and chromatin [31] and VDR-induced DNA methylation [32] (Fig. 3). These actions of the classical 1α,25-(OH)_2_ D_3_ genomic response modulate synthesis and accumulation of new proteins and invoke appropriate cellular responses, over intervals of hours or days. These events may also be suppressed by protein synthesis inhibitors, such as actinomycin D or cycloheximide [16].

### 1.7 1α,25-(OH)_2_ D_3_-mediated rapid or non-genomic responses

Notwithstanding the strong, non-covalent binding of 1α,25-(OH)_2_ D_3_ to its cognate receptor, this active sterol can also elicit rapid responses in target cells of the vitamin D endocrine system. Effects include generation of calcium or ion flux [33], induction of second messenger systems [34] and activation of cytosolic kinases [35]. 1α,25-(OH)_2_ D_3_ may promote rapid Ca^2+^ influx from the extracellular space through voltage-independent channels in rat osteosarcoma cells [36], release Ca^2+^ from intra-cellular stores in osteoblasts [37] and activate protein kinase pathways that may be Ca^2+^-dependent or -independent [37,38]. These effects occur rapidly within minutes and are considered incompatible with mechanisms involving alterations in gene transcription and protein synthesis [33]. Involvement of VDR in this pathway remains controversial, since rapid actions of 1α,25-(OH)_2_ D_3_ may be invoked in cells that lack VDR [39]. However, in various cell types rapid responses can be mediated by 1α,25-(OH)_2_ D_3_ acting through a population of classical VDR molecules acting outside the cell nucleus, associated with caveolae of the plasma membrane [40].

Importantly, analogs of different vitamin D isomers that are locked in *cis*- or *trans*-conformations have been synthesized which can preferentially elicit rapid non-genomic and/or genomic responses [9,10].

### 1.8 1α,25-(OH)_2_ D_3_ dual regulation of gene expression by genomic and rapid non-genomic pathways

There is compelling evidence that the rapid non-genomic activation of signal transduction pathways by 1α,25-(OH)_2_ D_3_ can interact with and modulate VDR-dependent gene transcription [41,42]. While the 1α,25-(OH)_2_ D_3_-liganded RXR–VDR heterocomplex selectively recognizes VDREs in the promoter regions of osteopontin (OPN) [43] and osteocalcin (OCN) [44] genes, the steady state levels of OPN and OCN mRNA can also be modulated by 1α,25-(OH)_2_ D_3_ rapid non-genomic actions [45]. Furthermore, antagonism of the non-genomic pathway blocks 1α,25-(OH)_2_ D_3_-mediated OCN expression [41]. 1α,25-(OH)_2_ D_3_ rapid activation of cytosolic kinases may phosphorylate critical coactivators resulting in modulation of VDR-dependent gene transcription [46]. By non-genomic actions, 1α,25-(OH)_2_ D_3_ can modulate a repertoire of cytosolic kinases and second messenger systems that show some level of cell- or tissue-specificity [38], e.g. activation of phospholipase A2 in chondrocytes [47] and protein kinase A in enterocytes [48]. By cross-talk with VDR/VDRE regulation of gene transcription, these membrane-mediated kinase cascades may influence cell-specific biological responses to 1α,25-(OH)_2_ D_3_, involved diverse physiological and pathobiological processes [7].

### 1.9 Tissue- and cell-specificity of vitamin D biological effects

Most tissues express the receptor for 1α,25-(OH)_2_ D_3_ (VDR) and renal tubules, skin, bone, brain, breast, colon and prostate also contain the enzyme CYP27B1, required for converting the major circulating metabolite of vitamin D, [25(OH)D] to 1α,25-(OH)_2_ D_3_ [49]. Notwithstanding the wide distribution of VDR and CYP27B1, 1α,25-(OH)_2_ D_3_ shows highly tissue-specific functional effects on hormone secretion, immune function, cell differentiation and growth. For example, 1α,25-(OH)_2_ D_3_ inhibits PTH secretion in the parathyroid glands [30] but stimulates pancreatic β-cell insulin secretion [50], inhibits adaptive immunity [51] but enhances some innate immune responses [52], inhibits differentiation of B lymphocytes [53] but enhances keratinocyte differentiation [54].

1α,25-(OH)_2_ D_3_-mediated growth effects may show similar cell-specificity. For example, 1α,25-(OH)_2_ D_3_ has antiproliferative effects in some neoplastic cells [55] but induces a spectrum of growth responses in others. The PC-3 and DU-145 prostate cancer cell lines for example are not significantly inhibited by physiologically relevant doses of 1α,25-(OH)_2_ D_3_ [56]. Furthermore, at low or physiological concentrations, 1α,25-(OH)_2_ D_3_ may promote proliferation of monocytes [57] or keratinocytes [58]. Effects upon anchorage-independent growth and invasion may show similar cell-specificity. For example, 1α,25-(OH)_2_ D_3_ or analogs inhibited anchorage-independent growth of prostate cancer cells [59] and suppressed invasion through Matrigel by these cells [59] and neuroblastoma cells [60]. Conversely, vitamin D or analogs may also enhance anchorage-independent growth [61] and promote 12-O-tetradecanoylphorbol-13-acetate (TPA) induced neoplastic transformation in JB6 epidermal cells [62].

These observations do not support uniform growth or other biological responses to vitamin D exposure. Rather, cell- or tissue-specific processes, could modulate the initiating 1α,25-(OH)_2_ D_3_ signal to provide different functional outcomes.

## 2 The Yin and Yang of 1α,25-(OH)_2_ D_3_ growth regulatory signaling

Expression profiling has identified diverse targets of 1α,25-(OH)_2_ D_3_ non-genomic or genomic actions including G-coupled receptors, inter- and intra-cellular signaling genes, cell-cycle regulators, metabolic function moeties, extracellular matrix components and cell adhesion molecules [63]. From within these networks, 1α,25-(OH)_2_ D_3_ not only regulates bone mineralization but also modulates growth and differentiation [64]. Such diversity of biological effect could be achieved in part through genomic/non-genomic cross-talk, molecular networks or transcriptomes implicated in lineage specialization or effects on target genes with context-specific functions.

Within such modular networks, osteopontin (OPN) and E-cadherin play important roles in growth responses to vitamin D [65,66]. Osteopontin (OPN) is a key vitamin D target gene, regulated by 1α,25-(OH)_2_ D_3_-mediated genomic [26] and non-genomic mechanisms [45,67]. OPN is an extracellular matrix glycophosphoprotein implicated in osteoblast differentiation [45] but is also a central effector of vitamin D – mediated anchorage-independent growth [68]. OPN may abrogate the adhesion requirement for cell growth and enhance cell invasion through Matrigel by activation of Ran GTPase (RAN) [69]. 1α,25-(OH)_2_ D_3_ transcriptional regulation of OPN involves VDR/RXR heterodimer binding and recruitment of coregulators including SRC-1, -2, -3, CBP, p300 and DRIP205 to VDREs within the OPN promoter [26]. Mutation at one or both VDRE sites in the rat OPN promoter substantively suppresses 1α,25-(OH)_2_ D_3_-mediated transcription of a OPN-promoter luciferase reporter construct [70]. However, OPN mRNA may also be activated by VDR non-genomic actions, involving Ca^2+^-influx and rapid activation of the small GTPAse, RhoA and its effector, Rho-associated coiled kinase (ROCK) [67]. Effects of this non-genomic pathway on OPN protein expression are unclear [67]. RhoA–ROCK activation of OPN mRNA in smooth muscle cells is Erk dependent and may be suppressed by the MEK1 inhibitor, PD98059 [71].

E-cadherin is induced by 1α,25-(OH)_2_ D_3_ non-genomic rapid actions [67] and suppresses cell growth, partly by inhibition of β-catenin transcriptional activity [72,73]. Free β-catenin that is sequestered by E-cadherin, is rapidly phosphorylated by glycogen synthase kinase3β (GSK3β) in the adenomatous polyposis coli (APC)/axin/GSK-3β/casein kinase I complex and is subsequently ubiquitinated and degraded. Loss of this function enables β-catenin accumulation and translocation to the nucleus where it modulates the expression of Tcf/Lef-1-target genes implicated in cell proliferation [74]. 1α,25-(OH)_2_ D_3_ induction of E-cadherin involves transcription-independent promotion of Ca^2+^-influx and consequent activation of RhoA–ROCK signaling. Subsequent to these events, induction of p38/MAPK-MSK1 signaling upregulates E-cadherin and inhibits β-catenin/Tcf transcriptional activity [67]. Hence, OPN and E-cadherin are functionally antagonistic growth regulatory genes that are modulated by 1α,25-(OH)_2_ D_3_ through overlapping but distinct molecular mechanisms.

### 2.1 Growth signaling through E-cadherin and OPN: functional outcomes of 1α,25-(OH)_2_ D_3_ treatment

E-cadherin and OPN are reciprocally regulated through β-catenin/Tcf and related signaling pathways [75] to provide high and low levels respectively, in quiescent normal tissue [76,77]. Disturbance of this equilibrium in early stages of multistep tumorigenesis may have phenotypic effects on cell adhesion [78], migration [76] and invasion [68]. Aberrant expression of these genes in early tumorigenesis may influence the subsequent development of abnormal molecular circuitry in evolving cancer cells.

To investigate involvement of the E-cadherin/OPN equilibrium in 1α,25-(OH)_2_ D_3_ growth control, Xu et al. used parental cell lines and stably transformed subclones with variable constitutive expression of these genes [70]. Parental R37 mammary cells highly express E-cadherin, have an epithelial-like morphology, have low levels of Tcf-1 indicative of low level β-catenin signaling activity and weak expression of OPN. Subclones were raised by stable transfection of R37 cells with metastatic tumor DNA fragments (R37 Met-DNA; [C9] cells) that upregulates OPN [79]. Subclones were also raised by transfection of parental R37 cells with OPN cDNA in sense or antisense orientations respectively, in expression vectors. R37 Met-DNA [C9] cells express high levels of OPN and Tcf-1, low levels of E-cadherin, have a spindle-like morphology and are invasive [75]. In these cells, OPN is considered to be the direct effector of Met-DNA, in promotion of invasion or metastasis [80]. In this model system, 1α,25-(OH)_2_ D_3_ and novel A-ring modified vitamin D analogs influenced the balance of these antagonistic VDR-dependent signals. All treatments upregulated E-cadherin, suppressed β-catenin transcriptional activity and β-catenin nuclear localization, consistent with growth-inhibition. However, all treatments also upregulated OPN that may be implicated in neoplastic transformation and invasion.

Although molecular cross-talk was observed, growth effects induced by 1α,25-(OH)_2_ D_3_ or analogs appeared dependent upon the constitutive balance of these functionally antagonistic molecules, in target cells. VDR ligands significantly increased migration or invasion only in those cells with high constitutive OPN and low E-cadherin [70]. This finding suggests that the pre-treatment activity state of antagonistic VDR-dependent molecules may influence cell-specific 1α,25-(OH)_2_ D_3_ growth responses. These fundamental studies may provide greater understanding of 1α,25-(OH)_2_ D_3_ growth effects relating to cancer, in whole animal studies as well as clinical or epidemiological surveys.

### 2.2 Tumorigenesis in VDR knockout mice

Development of a null mouse model has provided an important tool for study of 1α,25-(OH)_2_ D_3_/VDR functional roles in tumor biology. VDR null mice appear developmentally normal at birth but manifest growth abnormalities, alopecia and infertility from the time of weaning [81]. Mice deficient in VDR or key components of the vitamin D synthetic pathway do not manifest any increase of sporadic tumorigenesis [82]. However, VDR ablation alters susceptibility to chemically induced carcinogenesis in a tissue-specific manner [83]. Administration of DMBA together with medroxprogesterone induced increased formation of skin tumors and mammary hyperplasia in VDR knockout *vs*. control mice [83]. Although differences were observed in development of thymic lymphomas, lymphoblastic leukemias and mammary tumor histopathology, VDR status did not affect overall non-epidermal tumor incidence. No effects were observed on tumorigenesis in the ovary, uterus, liver or lung, despite expression of the VDR in these tissues. These findings provide strong evidence that VDR signaling alters chemically induced carcinogenesis in a manner that is tissue-specific but unrelated to VDR expression, in vivo [83].

### 2.3 Vitamin D exposures and human cancer risk

Associations between sunlight exposure, dietary histories and tumor incidence in various epidemiological surveys imply an important role for vitamin D in lifetime cancer risk and/or survival [84]. For example, mortality from colon cancer was found to be higher in geographical regions of the United States with low sunlight exposure [85] while decreased colon [86], breast [87] and prostate cancer risk [88] were reported in high sunlight areas. Inverse relationships between dietary vitamin D intake and breast cancer have been reported [89,90] although findings for colorectal cancer appeared inconsistent [91,92] and no clear associations were found for prostate cancer [93,94]. The relationship between sunlight exposure and risk of lymphoma has also been controversial. Studies have shown positive [95,96], inconsistent [97] or inverse [98] relationships between estimated solar ultraviolet exposure and non-Hodgkins lymphoma (NHL). No clear association between dietary intake of vitamin D and risk of NHL, diffuse large B-cell lymohoma, chronic lymphocytic leukemia or follicular lymphoma were found [99]. Study participants’ histories of sun exposure or intake of foods that are high in vitamin D may however be subject to systematic recall error and associated bias.

The vitamin D metabolite 25-OHD has a long half-life and serum levels may provide a robust biomarker of vitamin D status [100]. Subjects with high levels of 25-OHD were found to have a lower incidence of colorectal cancer (CRC), in both women and men [101,102], Freedman and colleagues confirmed an inverse association between serum 25-OHD levels and CRC risk and demonstrated that the highest percentile serum 25-OHD levels had a CRC relative risk (RR) of 0.28 [103]. No clear association with total cancer mortality was observed, however [103]. Associations between 25-OHD levels, breast [104,105] and total prostate cancer [106–108] have been inconsistent. In a nested case–control study involving 270 incident lymphoid cancer cases and 538 controls from a cohort of 29,133 Finnish male smokers, serum 25-OHD levels were not associated with the risk of overall lymphoid cancers, NHL or multiple myeloma [109]. However, high serum serum 25-OHD levels were associated with increased risk of pancreatic cancer in 200 Finnish cases and 400 controls, from this same total cohort [110]. In a further nested case–control study, high prediagnostic serum 25-OHD levels had increased risk of aggressive prostate cancer in 749 cases and 781 control subjects from the US Prostate, Lung, Colorectal and Ovarian (PLCO) Cancer Screening Trial [108].

In the nested case–control study design, non-diseased subjects from whom the controls are selected may not be fully representative of the healthy population [111]. In a prospective cohort study of >700 apparently healthy adults from the Linxian region of China, higher serum 25(OH)D concentrations were associated with significantly increased risk of development of esophageal squamous dysplasia [112] and invasive squamous carcinoma [113].

These epidemiological studies show positive and negative associations between vitamin D status and common solid cancers in various populations and allude to tissue-specificity of effect. At present, any chemopreventive benefits of higher serum 25(OH)D levels for colorectal cancer [102,103] may require to be weighed against potential increased risks of esophageal squamous dysplasia [112] or cancer [113], pancreatic cancer [110] or aggressive prostate cancer [108]. Within interacting molecular networks that influence tissue-specific responses to 1α,25-(OH)_2_ D_3_, a dynamic equilibrium of positive or negative growth signals may determine ultimate outcomes, although key molecules can have specific effects upon development of neoplastic phenotype.

### 2.4 Tissue-specific VDR operational networks and cancer risk

The VDR growth regulatory equilibrium involving E-cadherin and OPN is disturbed during stepwise evolution of many human cancers [77,114] although there are important tissue-specific differences. In the colon for example, adenomatous polyps represent the commonest identifiable premalignant lesion. These colonic adenomas have E-cadherin/OPN expression patterns resembling that of normal mucosa, namely preservation of E-cadherin [115] and low or undetectable OPN [116]. Hence, in these lesions, high level serum [25(OH)D] could further increase E-cadherin tumor suppressor activity, set against low level OPN with an overall effect of growth restraint. By this rationale, persistently high serum [25(OH)D] could impede neoplastic progression of colonic adenomas which are common in asymptomatic Western populations [117], with ultimate reduction of CRC incidence. Conversely, preneoplastic squamous mucosal hyperplasia or dysplasia are common in high risk areas for esophageal cancer [118]. Unlike colonic adenomas, these lesions are characterised by early suppression of E-cadherin [119] and upregulation of OPN [120]. In such populations, higher serum [25(OH)D] levels are associated with increased esophageal squamous cancer risk [113]. Potentially, high 1α,25-(OH)_2_ D_3_ exposure could enhance the predominance of OPN growth-promoting signals and associated Ran activity [69] in these lesions, beyond the threshold level required for invasion and neoplastic progression. Neoplasms of the pancreas and prostate are characterised by similar disequilibrium involving low E-cadherin and high OPN [121–124] which could potentially be related to the direct associations between serum [25(OH)D] levels and risks of pancreatic cancer or aggressive prostate cancer [67,108,110].

Tissue-specific 1α,25-(OH)_2_ D_3_ growth responses may involve complex non-genomic/genomic cross-talk and modulation of downstream signals by distinct lineage-specific expression patterns of the human transcriptome. However, the E-cadherin/OPN expression balance may provide a useful biologically based marker of this complexity, implicated in 1α,25-(OH)_2_ D_3_ growth responses.

### 2.5 Towards conditional targeting strategies

The above studies address fundamental mechanisms and link tissue-specific differences of VDR growth regulatory networks, particularly involving OPN and E-cadherin to epidemiological associations between serum [25(OH)D] levels and cancer.

Although genomic effects of 1α,25-(OH)_2_ D_3_ are partly implicated in induction of OPN [7], they appear dispensible for induction of E-cadherin in colorectal cells [67]. 1α,25-(OH)_2_ D_3_ induces transcription-independent Ca^2+^ influx and activation of RhoA-ROCK, in the presence VDR. Thus activated, RhoA-ROCK upregulates p38 MAPK and MSK1, leading to substantive induction of E-cadherin mRNA and protein, inhibition of β-catenin/Tcf transcriptional activity and suppression of cell proliferation [67]. OPN mRNA may also be regulated by 1α,25-(OH)_2_ D_3_ through transcription-independent, Ca^2+^-dependent RhoA/ROCK activation although the response appears less robust than that of E-cadherin. 1α,25-(OH)_2_ D_3_ induced 30-fold induction of E-cadherin mRNA but less than 10-fold upregulation of OPN mRNA within 8 h [67]. Downstream of Rho/ROCK, p38/MSK1 is required for induction of E-cadherin [67]. Conversely, in smooth muscle cells RhoA/ROCK activation of ERK is implicated in upregulation of OPN mRNA.

Additional work is required to explore mechanistic issues involving 1α,25-(OH)_2_ D_3_ rapid *vs*. genomic effects, regulation of signaling kinases and differential activation of E-cadherin/OPN growth regulatory genes. Rational design of combination therapies that allows activation of E-cadherin without upregulation of OPN may be a useful target for tissue-specific pharmacotherapeutics, particularly for preneoplastic states characterised by high constitutive OPN. Such future advances may provide a rationale for improved prevention and treatment of different cancers, through VDR mediated growth control.

## References

[1] Mellanby E. (1919). An experimental investigation on rickets. Lancet.

[2] Chick H., Dalyell E.J.H., Hume E.M., Mackay H.M.M., Henderson-Smith H. (1922). The aetiology of rickets in infants: prophylactic and curative observations at the Vienna University Kinderklinik. Lancet.

[3] Farber E. (1953). Nobel prize winners in chemistry.

[4] Holick M.F. (2004). Sunlight and vitamin D for bone health and prevention of autoimmune diseases, cancers, and cardiovascular disease. Am J Clin Nutr.

[5] DeLuca H.F. (2004). Overview of general physiologic features and functions of vitamin D. Am J Clin Nutr.

[6] Bouillon R., Carmeliet G., Verlinden L., van Etten E., Verstuyf A., Luderer H.F. (2008). Vitamin D and human health: lessons from vitamin D receptor null mice. Endocr Rev.

[7] Norman A.W. (2006). Minireview: vitamin D receptor: new assignments for an already busy receptor. Endocrinology.

[8] Haussler M.R., Haussler C.A., Jurutka P.W., Thompson P.D., Hsieh J.C., Remus L.S. (1997). The vitamin D hormone and its nuclear receptor: molecular actions and disease states. J Endocrinol.

[9] Posner G.H., Lee J.K., White M.C., Hutchings R.H., Dai H., Kachinski J.L. (1997). Antiproliferative hybrid analogs of the hormone 1alpha,25-dihydroxyvitamin D (3): design, synthesis, and preliminary biological evaluation. J Org Chem.

[10] Norman A.W., Song X., Zanello L., Bula C., Okamura W.H. (1999). Rapid and genomic biological responses are mediated by different shapes of the agonist steroid hormone, 1alpha, 25(OH)_2_ vitamin D3. Steroids.

[11] Arnaud J., Constans J. (1993). Affinity differences for vitamin D metabolites associated with the genetic isoforms of the human serum carrier protein (DBP). Hum Genet.

[12] Bikle D.D., Gee E., Halloran B., Kowalski M.A., Ryzen E., Haddad J.G. (1986). Assessment of the free fraction of 25-hydroxyvitamin D in serum and its regulation by albumin and the vitamin D-binding protein. J Clin Endocrinol Metab.

[13] Bikle D.D., Gee E. (1989). Free, and not total, 1,25-dihydroxyvitamin D regulates 25-hydroxyvitamin D metabolism by keratinocytes. Endocrinology.

[14] Safadi F.F., Thornton P., Magiera H., Hollis B.W., Gentile M., Haddad J.G. (1999). Osteopathy and resistance to vitamin D toxicity in mice null for vitamin D binding protein. J Clin Invest.

[15] Holick M.F., Holick M. (1999). Molecular recognition and structure–activity relations. Vitamin D: physiology, molecular biology, and clinical applications.

[16] Norman A.W., Mizwicki M.T., Norman D.P. (2004). Steroid-hormone rapid actions, membrane receptors and a conformational ensemble model. Nat Rev Drug Discov.

[17] Makishima M., Lu T.T., Xie W., Whitfield G.K., Domoto H., Evans R.M. (2002). Vitamin D receptor as an intestinal bile acid sensor. Science.

[18] Rochel N., Wurtz J.M., Mitschler A., Klaholz B., Moras D. (2000). The crystal structure of the nuclear receptor for vitamin D bound to its natural ligand. Mol Cell.

[19] Freedman L.P. (1999). Increasing the complexity of coactivation in nuclear receptor signaling. Cell.

[20] Mizwicki M.T., Bula C.M., Bishop J.E., Norman A.W. (2007). New insights into Vitamin D sterol-VDR proteolysis, allostery, structure-function from the perspective of a conformational ensemble model. J Steroid Biochem Mol Biol.

[21] Carlberg C., Norman A.W., Bouillon R., Thmasset M. (1997). Critical analysis of 1alpha 25-dihydroxy vitamin D3 response elements. Proceedings of the 10th international vitamin D workshop.

[22] Carlberg C. (1995). Mechanisms of nuclear signalling by vitamin D3. Interplay with retinoid and thyroid hormone signalling. Eur J Biochem.

[23] Toell A., Polly P., Carlberg C. (2000). All natural DR3-type vitamin D response elements show a similar functionality in vitro. Biochem J.

[24] McKenna N.J., O’Malley B.W. (2002). Combinatorial control of gene expression by nuclear receptors and coregulators. Cell.

[25] Torchia J., Glass C., Rosenfeld M.G. (1998). Co-activators and co-repressors in the integration of transcriptional responses. Curr Opin Cell Biol.

[26] Kim S., Shevde N.K., Pike J.W. (2005). 1,25-Dihydroxyvitamin D3 stimulates cyclic vitamin D receptor/retinoid X receptor DNA-binding, co-activator recruitment, and histone acetylation in intact osteoblasts. J Bone Miner Res.

[27] Spencer T.E., Jenster G., Burcin M.M., Allis C.D., Zhou J., Mizzen C.A. (1997). Steroid receptor coactivator-1 is a histone acetyltransferase. Nature.

[28] Norman A.W. (1971). Evidence for a new kidney-produced hormone, 1,25-dihydroxycholecalciferol, the proposed biologically active form of vitamin D. Am J Clin Nutr.

[29] Townsend K., Evans K.N., Campbell M.J., Colston K.W., Adams J.S., Hewison M. (2005). Biological actions of extra-renal 25-hydroxyvitamin D-1alpha-hydroxylase and implications for chemoprevention and treatment. J Steroid Biochem Mol Biol.

[30] Demay M.B., Kiernan M.S., DeLuca H.F., Kronenberg H.M. (1992). Sequences in the human parathyroid hormone gene that bind the 1,25-dihydroxyvitamin D3 receptor and mediate transcriptional repression in response to 1,25-dihydroxyvitamin D3. Proc Natl Acad Sci USA.

[31] Fujiki R., Kim M.S., Sasaki Y., Yoshimura K., Kitagawa H., Kato S. (2005). Ligand-induced transrepression by VDR through association of WSTF with acetylated histones. EMBO J.

[32] Kim M.S., Fujiki R., Kitagawa H., Kato S. (2007). 1alpha,25(OH)_2_ D_3_-induced DNA methylation suppresses the human CYP27B1 gene. Mol Cell Endocrinol.

[33] Cancela L., Nemere I., Norman A.W. (1988). 1 alpha,25(OH)2 vitamin D3: a steroid hormone capable of producing pleiotropic receptor-mediated biological responses by both genomic and nongenomic mechanisms. J Steroid Biochem.

[34] Farach-Carson M.C., Ridall A.L. (1998). Dual 1,25-dihydroxyvitamin D3 signal response pathways in osteoblasts: cross-talk between genomic and membrane-initiated pathways. Am J Kidney Dis.

[35] Boyan B.D., Schwartz Z. (2004). Rapid vitamin D-dependent PKC signaling shares features with estrogen-dependent PKC signaling in cartilage and bone. Steroids.

[36] Caffrey J.M., Farach-Carson M.C. (1989). Vitamin D3 metabolites modulate dihydropyridine-sensitive calcium currents in clonal rat osteosarcoma cells. J Biol Chem.

[37] Le Mellay V., Grosse B., Lieberherr M. (1997). Phospholipase C beta and membrane action of calcitriol and estradiol. J Biol Chem.

[38] Norman A.W., Okamura W.H., Bishop J.E., Henry H.L. (2002). Update on biological actions of 1alpha,25(OH)2-vitamin D3 (rapid effects) and 24R,25(OH)2-vitamin D3. Mol Cell Endocrinol.

[39] Wali R.K., Kong J., Sitrin M.D., Bissonnette M., Li Y.C. (2003). Vitamin D receptor is not required for the rapid actions of 1,25-dihydroxyvitamin D3 to increase intracellular calcium and activate protein kinase C in mouse osteoblasts. J Cell Biochem.

[40] Huhtakangas J.A., Olivera C.J., Bishop J.E., Zanello L.P., Norman A.W. (2004). The vitamin D receptor is present in caveolae-enriched plasma membranes and binds 1 alpha 25(OH)2-vitamin D3 in vivo and in vitro. Mol Endocrinol.

[41] Baran D.T., Sorensen A.M., Shalhoub V., Owen T., Stein G., Lian J. (1992). The rapid nongenomic actions of 1 alpha 25-dihydroxyvitamin D3 modulate the hormone-induced increments in osteocalcin gene transcription in osteoblast-like cells. J Cell Biochem.

[42] Sitrin M.D., Bissonnette M., Bolt M.J., Wali R., Khare S., Scaglione-Sewell B. (1999). Rapid effects of 1,25(OH)_2_ vitamin D3 on signal transduction systems in colonic cells. Steroids.

[43] Noda M., Vogel R.L., Craig A.M., Prahl J., DeLuca H.F., Denhardt D.T. (1990). Identification of a DNA sequence responsible for binding of the 1,25-dihydroxyvitamin D3 receptor and 1,25-dihydroxyvitamin D3 enhancement of mouse secreted phosphoprotein 1 (SPP-1 or osteopontin) gene expression. Proc Natl Acad Sci USA.

[44] MacDonald P.N., Haussler C.A., Terpening C.M., Galligan M.A., Reeder M.C., Whitfield G.K. (1991). Baculovirus-mediated expression of the human vitamin D receptor. Functional characterization, vitamin D response element interactions, and evidence for a receptor auxiliary factor. J Biol Chem.

[45] Jenis L.G., Lian J.B., Stein G.S., Baran D.T. (1993). 1 alpha, 25-dihydroxyvitamin D3-induced changes in intracellular pH in osteoblast-like cells modulate gene expression. J Cell Biochem.

[46] Barletta F., Freedman L.P., Christakos S. (2002). Enhancement of VDR-mediated transcription by phosphorylation: correlation with increased interaction between the VDR and DRIP205, a subunit of the VDR-interacting protein coactivator complex. Mol Endocrinol.

[47] Boyan B.D., Wang L., Wong K.L., Jo H., Schwartz Z. (2006). Plasma membrane requirements for 1alpha, 25(OH)_2_ D_3_ dependent PKC signaling in chondrocytes and osteoblasts. Steroids.

[48] Nemere I. (1999). 24,25-Dihydroxyvitamin D3 suppresses the rapid actions of 1, 25-dihydroxyvitamin D3 and parathyroid hormone on calcium transport in chick intestine. J Bone Miner Res.

[49] Anderson P.H., Hendrix I., Sawyer R.K., Zarrinkalam R., Manavis J., Sarvestani G.T. (2008). Co-expression of CYP27B1 enzyme with the 1.5 kb CYP27B1 promoter-luciferase transgene in the mouse. Mol Cell Endocrinol.

[50] Lee S., Clark S.A., Gill R.K., Christakos S. (1994). 1,25-Dihydroxyvitamin D3 and pancreatic beta-cell function: vitamin D receptors, gene expression, and insulin secretion. Endocrinology.

[51] Lemire J.M., Archer D.C., Beck L., Spiegelberg H.L. (1995). Immunosuppressive actions of 1,25-dihydroxyvitamin D3: preferential inhibition of Th1 functions. J Nutr.

[52] Wang T.T., Nestel F.P., Bourdeau V., Nagai Y., Wang Q., Liao J. (2004). Cutting edge: 1,25-dihydroxyvitamin D3 is a direct inducer of antimicrobial peptide gene expression. J Immunol.

[53] Chen S., Sims G.P., Chen X.X., Gu Y.Y., Chen S., Lipsky P.E. (2007). Modulatory effects of 1,25-dihydroxyvitamin D3 on human B cell differentiation. J Immunol.

[54] Bikle D.D., Pillai S. (1993). Vitamin D, calcium, and epidermal differentiation. Endocr Rev.

[55] Banerjee P., Chatterjee M. (2003). Antiproliferative role of vitamin D and its analogs—a brief overview. Mol Cell Biochem.

[56] Campbell M.J., Gombart A.F., Kwok S.H., Park S., Koeffler H.P. (2000). The anti-proliferative effects of 1alpha, 25(OH)_2_ D_3_ on breast and prostate cancer cells are associated with induction of BRCA1 gene expression. Oncogene.

[57] Ohta M., Okabe T., Ozawa K., Urabe A., Takaku F. (1985). 1 alpha,25-Dihydroxyvitamin D3 (calcitriol) stimulates proliferation of human circulating monocytes in vitro. FEBS Lett.

[58] Itin P.H., Pittelkow M.R., Kumar R. (1994). Effects of vitamin D metabolites on proliferation and differentiation of cultured human epidermal keratinocytes grown in serum-free or defined culture medium. Endocrinology.

[59] Tokar E.J., Webber MM (2005). Cholecalciferol (vitamin D3) inhibits growth and invasion by up-regulating nuclear receptors and 25-hydroxylase (CYP27A1) in human prostate cancer cells. Clin Exp Metastasis.

[60] Reddy C.D., Patti R., Guttapalli A., Maris J.M., Yanamandra N., Rachamallu A. (2006). Anticancer effects of the novel 1alpha, 25-dihydroxyvitamin D3 hybrid analog QW1624F2-2 in human neuroblastoma. J Cell Biochem.

[61] Hosoi J., Abe E., Suda T., Colburn N.H., Kuroki T. (1986). Induction of anchorage-independent growth of JB6 mouse epidermal cells by 1 alpha,25-dihydroxyvitamin D3. Cancer Res.

[62] Chang P.L., Lee T.F., Garretson K., Prince C.W. (1997). Calcitriol enhancement of TPA-induced tumorigenic transformation is mediated through vitamin D receptor-dependent and -independent pathways. Clin Exp Metastasis.

[63] Wood R.J., Tchack L., Angelo G., Pratt R.E., Sonna L.A. (2004). DNA microarray analysis of vitamin D-induced gene expression in a human colon carcinoma cell line. Physiol Genomics.

[64] Colston K.W., Hansen C.M. (2002). Mechanisms implicated in the growth regulatory effects of vitamin D in breast cancer. Endocr Relat Cancer.

[65] Chang P.L., Prince C.W. (1993). 1 alpha,25-Dihydroxyvitamin D3 enhances 12-O-tetradecanoylphorbol-13-acetate- induced tumorigenic transformation and osteopontin expression in mouse JB6 epidermal cells. Cancer Res.

[66] Palmer H.G., Gonzalez-Sancho J.M., Espada J., Berciano M.T., Puig I., Baulida J. (2001). Vitamin D(3) promotes the differentiation of colon carcinoma cells by the induction of E-cadherin and the inhibition of beta-catenin signaling. J Cell Biol.

[67] Ordonez-Moran P., Larriba M.J., Palmer H.G., Valero R.A., Barbachano A., Dunach M. (2008). RhoA-ROCK and p38MAPK-MSK1 mediate vitamin D effects on gene expression, phenotype, and Wnt pathway in colon cancer cells. J Cell Biol.

[68] Tuck A.B., Arsenault D.M., O’Malley F.P., Hota C., Ling M.C., Wilson S.M. (1999). Osteopontin induces increased invasiveness and plasminogen activator expression of human mammary epithelial cells. Oncogene.

[69] Kurisetty V.V., Johnston P.G., Johnston N., Erwin P., Crowe P., Fernig D.G. (2008). RAN GTPase is an effector of the invasive/metastatic phenotype induced by osteopontin. Oncogene.

[70] Xu H., McCann M., Zhang Z., Posner G.H., Bingham V., El-Tanani M. (2009). Vitamin D receptor modulates the neoplastic phenotype through antagonistic growth regulatory signals. Mol Carcinog.

[71] Kawamura H., Yokote K., Asaumi S., Kobayashi K., Fujimoto M., Maezawa Y. (2004). High glucose-induced upregulation of osteopontin is mediated via Rho/Rho kinase pathway in cultured rat aortic smooth muscle cells. Arterioscler Thromb Vasc Biol.

[72] Stockinger A., Eger A., Wolf J., Beug H., Foisner R. (2001). E-cadherin regulates cell growth by modulating proliferation-dependent beta-catenin transcriptional activity. J Cell Biol.

[73] Shah S., Islam M.N., Dakshanamurthy S., Rizvi I., Rao M., Herrell R. (2006). The molecular basis of vitamin D receptor and beta-catenin crossregulation. Mol Cell.

[74] Nelson W.J., Nusse R. (2004). Convergence of Wnt, beta-catenin, and cadherin pathways. Science.

[75] El-Tanani M.K., Barraclough R., Wilkinson M.C., Rudland P.S. (2001). Regulatory region of metastasis-inducing DNA is the binding site for T cell factor-4. Oncogene.

[76] Gumbiner B.M. (1996). Cell adhesion: the molecular basis of tissue architecture and morphogenesis. Cell.

[77] Coppola D., Szabo M., Boulware D., Muraca P., Alsarraj M., Chambers A.F. (2004). Correlation of osteopontin protein expression and pathological stage across a wide variety of tumor histologies. Clin Cancer Res.

[78] Gottardi C.J., Wong E., Gumbiner B.M. (2001). E-cadherin suppresses cellular transformation by inhibiting beta-catenin signaling in an adhesion-independent manner. J Cell Biol.

[79] El-Tanani M., Barraclough R., Wilkinson M.C., Rudland P.S. (2001). Metastasis-inducing DNA regulates the expression of the osteopontin gene by binding the transcription factor Tcf-4. Cancer Res.

[80] Moye V.E., Barraclough R., West C., Rudland P.S. (2004). Osteopontin expression correlates with adhesive and metastatic potential in metastasis-inducing DNA-transfected rat mammary cell lines. Br J Cancer.

[81] Yoshizawa T., Handa Y., Uematsu Y., Takeda S., Sekine K., Yoshihara Y. (1997). Mice lacking the vitamin D receptor exhibit impaired bone formation, uterine hypoplasia and growth retardation after weaning. Nat Genet.

[82] Panda D.K., Miao D., Tremblay M.L., Sirois J., Farookhi R., Hendy G.N. (2001). Targeted ablation of the 25-hydroxyvitamin D 1alpha-hydroxylase enzyme: evidence for skeletal, reproductive, and immune dysfunction. Proc Natl Acad Sci USA.

[83] Zinser G.M., Suckow M., Welsh J. (2005). Vitamin D receptor (VDR) ablation alters carcinogen-induced tumorigenesis in mammary gland, epidermis and lymphoid tissues. J Steroid Biochem Mol Biol.

[84] Giovannucci E. (2005). The epidemiology of vitamin D and cancer incidence and mortality: a review (United States). Cancer Causes Control.

[85] Garland C.F., Garland F.C. (1980). Do sunlight and vitamin D reduce the likelihood of colon cancer?. Int J Epidemiol.

[86] Robsahm T.E., Tretli S., Dahlback A., Moan J. (2004). Vitamin D3 from sunlight may improve the prognosis of breast-, colon- and prostate cancer (Norway). Cancer Causes Control.

[87] Garland F.C., Garland C.F., Gorham E.D., Young J.F. (1990). Geographic variation in breast cancer mortality in the United States: a hypothesis involving exposure to solar radiation. Prev Med.

[88] John E.M., Koo J., Schwartz G.G. (2007). Sun exposure and prostate cancer risk: evidence for a protective effect of early-life exposure. Cancer Epidemiol Biomarkers Prev.

[89] John E.M., Schwartz G.G., Dreon D.M., Koo J. (1999). Vitamin D and breast cancer risk: the NHANES I epidemiologic follow-up study, 1971–1975 to 1992. National Health and Nutrition Examination Survey. Cancer Epidemiol Biomarkers Prev.

[90] Knight J.A., Lesosky M., Barnett H., Raboud J.M., Vieth R. (2007). Vitamin D and reduced risk of breast cancer: a population-based case–control study. Cancer Epidemiol Biomarkers Prev.

[91] Garland C., Shekelle R.B., Barrett-Connor E., Criqui M.H., Rossof A.H., Paul O. (1985). Dietary vitamin D and calcium and risk of colorectal cancer: a 19-year prospective study in men. Lancet.

[92] Levi F., Pasche C., Lucchini F., La Vecchia C. (2000). Selected micronutrients and colorectal cancer. A case–control study from the canton of Vaud, Switzerland. Eur J Cancer.

[93] Kristal A.R., Cohen J.H., Qu P., Stanford J.L. (2002). Associations of energy, fat, calcium, and vitamin D with prostate cancer risk. Cancer Epidemiol Biomarkers Prev.

[94] Tseng M., Breslow R.A., Graubard B.I., Ziegler R.G. (2005). Dairy, calcium, and vitamin D intakes and prostate cancer risk in the National Health and Nutrition Examination Epidemiologic Follow-up Study cohort. Am J Clin Nutr.

[95] Bentham G. (1996). Association between incidence of non-Hodgkin's lymphoma and solar ultraviolet radiation in England and Wales. Br Med J.

[96] Zhang Y., Holford T.R., Leaderer B., Boyle P., Zhu Y., Wang R. (2007). Ultraviolet radiation exposure and risk of non-Hodgkin's lymphoma. Am J Epidemiol.

[97] Hartge P., Devesa S.S., Grauman D., Fears T.R., Fraumeni J.F. (1996). Non-Hodgkin's lymphoma and sunlight. J Natl Cancer Inst.

[98] Hughes A.M., Armstrong B.K., Vajdic C.M., Turner J., Grulich A.E., Fritschi L. (2004). Sun exposure may protect against non-Hodgkin lymphoma: a case–control study. Int J Cancer.

[99] Chang E.T., Balter K.M., Torrang A., Smedby K.E., Melbye M., Sundstrom C. (2006). Nutrient intake and risk of non-Hodgkin's lymphoma. Am J Epidemiol.

[100] Bouillon R., Okamura W.H., Norman A.W. (1995). Structure-function relationships in the vitamin D endocrine system. Endocr Rev.

[101] Feskanich D., Ma J., Fuchs C.S., Kirkner G.J., Hankinson S.E., Hollis B.W. (2004). Plasma vitamin D metabolites and risk of colorectal cancer in women. Cancer Epidemiol Biomarkers Prev.

[102] Tangrea J., Helzlsouer K., Pietinen P., Taylor P., Hollis B., Virtamo J. (1997). Serum levels of vitamin D metabolites and the subsequent risk of colon and rectal cancer in Finnish men. Cancer Causes Control.

[103] Freedman D.M., Looker A.C., Chang S.C., Graubard B.I. (2007). Prospective study of serum vitamin D and cancer mortality in the United States. J Natl Cancer Inst.

[104] Bertone-Johnson E.R., Chen W.Y., Holick M.F., Hollis B.W., Colditz G.A., Willett W.C. (2005). Plasma 25-hydroxyvitamin D and 1, 25-dihydroxyvitamin D and risk of breast cancer. Cancer Epidemiol Biomarkers Prev.

[105] Hiatt R.A., Krieger N., Lobaugh B., Drezner M.K., Vogelman J.H., Orentreich N. (1998). Prediagnostic serum vitamin D and breast cancer. J Natl Cancer Inst.

[106] Ahonen M.H., Tenkanen L., Teppo L., Hakama M., Tuohimaa P. (2000). Prostate cancer risk and prediagnostic serum 25-hydroxyvitamin D levels (Finland). Cancer Causes Control.

[107] Corder E.H., Guess H.A., Hulka B.S., Friedman G.D., Sadler M., Vollmer R.T. (1993). Vitamin D and prostate cancer: a prediagnostic study with stored sera. Cancer Epidemiol Biomarkers Prev.

[108] Ahn J., Peters U., Albanes D., Purdue M.P., Abnet C.C., Chatterjee N. (2008). Serum vitamin D concentration and prostate cancer risk: a nested case–control study. J Natl Cancer Inst.

[109] Lim U., Freedman D.M., Hollis B.W., Horst R.L., Purdue M.P., Chatterjee N. (2009). A prospective investigation of serum 25-hydroxyvitamin D and risk of lymphoid cancers. Int J Cancer.

[110] Stolzenberg-Solomon R.Z., Vieth R., Azad A., Pietinen P., Taylor P.R., Virtamo J. (2006). A prospective nested case–control study of vitamin D status and pancreatic cancer risk in male smokers. Cancer Res.

[111] Ernster V.L. (1994). Nested case–control studies. Prev Med.

[112] Abnet C.C., Chen W., Dawsey S.M., Wei W.Q., Roth M.J., Liu B. (2007). Serum 25(OH)-vitamin D concentration and risk of esophageal squamous dysplasia. Cancer Epidemiol Biomarkers Prev.

[113] Chen W., Dawsey S.M., Qiao Y.L., Mark S.D., Dong Z.W., Taylor P.R. (2007). Prospective study of serum 25(OH)-vitamin D concentration and risk of oesophageal and gastric cancers. Br J Cancer.

[114] Christofori G., Semb H. (1999). The role of the cell-adhesion molecule E-cadherin as a tumour-suppressor gene. Trends Biochem Sci.

[115] van der Wurff A.A., Vermeulen S.J., van der Linden E.P., Mareel M.M., Bosman F.T., Arends J.W. (1997). Patterns of alpha- and beta-catenin and E-cadherin expression in colorectal adenomas and carcinomas. J Pathol.

[116] Rohde F., Rimkus C., Friederichs J., Rosenberg R., Marthen C., Doll D. (2007). Expression of osteopontin, a target gene of de-regulated Wnt signaling, predicts survival in colon cancer. Int J Cancer.

[117] Pinsky P.F., Schoen R.E., Weissfeld J.L., Church T., Yokochi L.A., Doria-Rose V.P. (2008). The yield of surveillance colonoscopy by adenoma history and time to examination. Clin Gastroenterol Hepatol.

[118] Wei W.Q., Abnet C.C., Lu N., Roth M.J., Wang G.Q., Dye B.A. (2005). Risk factors for oesophageal squamous dysplasia in adult inhabitants of a high risk region of China. Gut.

[119] Santos-Garcia A., Abad-Hernandez M.M., Fonseca-Sanchez E., Julian-Gonzalez R., Galindo-Villardon P., Cruz-Hernandez J.J. (2006). E-cadherin, laminin and collagen IV expression in the evolution from dysplasia to oral squamous cell carcinoma. Med Oral Patol Oral Cir Bucal.

[120] Devoll R.E., Li W., Woods K.V., Pinero G.J., Butler W.T., Farach-Carson M.C. (1999). (OPN) distribution in premalignant and malignant lesions of oral epithelium and expression in cell lines derived from squamous cell carcinoma of the oral cavity. J Oral Pathol Med.

[121] Van Heek N.T., Maitra A., Koopmann J., Fedarko N., Jain A., Rahman A. (2004). Gene expression profiling identifies markers of ampullary adenocarcinoma. Cancer Biol Ther.

[122] Perl A.K., Wilgenbus P., Dahl U., Semb H., Christofori G. (1998). A causal role for E-cadherin in the transition from adenoma to carcinoma. Nature.

[123] Rubin M.A., Mucci N.R., Figurski J., Fecko A., Pienta K.J., Day M.L. (2001). E-cadherin expression in prostate cancer: a broad survey using high-density tissue microarray technology. Hum Pathol.

[124] Castellano G., Malaponte G., Mazzarino M.C., Figini M., Marchese F., Gangemi P. (2008). Activation of the osteopontin/matrix metalloproteinase-9 pathway correlates with prostate cancer progression. Clin Cancer Res.

[125] Yamada S., Yamamoto K., Masuno H., Choi M. (2001). Three-dimensional structure–function relationship of vitamin D and vitamin D receptor model. Steroids.

[126] Deeb K.K., Trump D.L., Johnson C.S., Vitamin (2007). D signalling pathways in cancer: potential for anticancer therapeutics. Nat Rev Cancer.

